# Results of the Modified Brosch Approach and Rehabilitation of Mural Unicystic Ameloblastoma of the Mandible in a Young Patient

**DOI:** 10.7759/cureus.36441

**Published:** 2023-03-20

**Authors:** Vijayanirmala Subramani, Giri Govindarajan Valandhan Vedha, Preethi Vijayarani

**Affiliations:** 1 Oral Pathology and Microbiology, Sri Ramachandra Dental College and Hospital, Sri Ramachandra Institute of Higher Education and Research, Chennai, IND; 2 Oral and Maxillofacial Surgery, Sri Ramachandra Dental College and Hospital, Sri Ramachandra Institute of Higher Education and Research, Chennai, IND

**Keywords:** good bone regeneration, quality of life, esthetic, conservative approach, low recurrence

## Abstract

Although radical surgery results in craniofacial deformity, functional damage, and aesthetic harm, all of which have an immediate negative impact on a patient’s quality of life, it also has the lowest recurrence rate for biologically aggressive subtypes of mural unicystic ameloblastomas. The oral approach removes the lingual cortex and exposes the entire buccal cortical plate in the area of the ascending and horizontal ramus to the full extent of the lesion. There is no obvious distortion of the soft tissues of the face following the modified Brosch procedure, which is also associated with rapid bone regeneration. This approach resulted in less invasive surgery and low surgical morbidity and recurrence in a young adult with a large mural unicystic ameloblastoma.

## Introduction

Unicystic ameloblastomas typically develop in the second decade of life and are linked to an impacted tooth. Their clinical and histological features influence the aggressiveness of a unicystic ameloblastoma, which, in turn, affects treatment and recurrence [1,2]. The treatment modalities with the lowest recurrence rate of unicystic ameloblastoma include enucleation alone (30.5%), enucleation followed by Carnoy’s solution (16%), marsupialization (18%), and resection. Lau et al. conducted a comprehensive review of treatment options for unicystic ameloblastomas and advised a more cautious approach with a low recurrence rate and little surgical tissue loss. The Brosch procedure involves the removal of the lateral cortical plate through a transoral approach, enabling the surgeon to visualize and completely enucleate the cyst. It also facilitates the treatment of the remaining bone and the excision of affected areas of the overlying soft tissue. The modified Brosch method allows the affected medial cortex and any perforations to be removed. With this background [3], this paper presents a thorough application and customization of a mandibular mural unicystic ameloblastoma that was treated with a modified Brosch technique and Carnoy’s solution, followed by postoperative rehabilitation.

## Case presentation

A young adult in the second decade of life had been experiencing puffiness with pain in the lower right back region of the mandible. The swelling had started out slowly before growing to its current extent. He reported mild and intermittent pain. On clinical examination, extraoral tenderness was present in relation to the right inferior border of the mandible. On intraoral examination, a firm bony mass approximately 7 cm × 3.5 cm in size and extending anteroposteriorly from the distal position of 46 extending to the sigmoid notch region and superoinferiorly extending from the sigmoid notch to the lower buccal and lingual vestibule and vestibules tenderness was noted on palpation.

**Figure 1:**
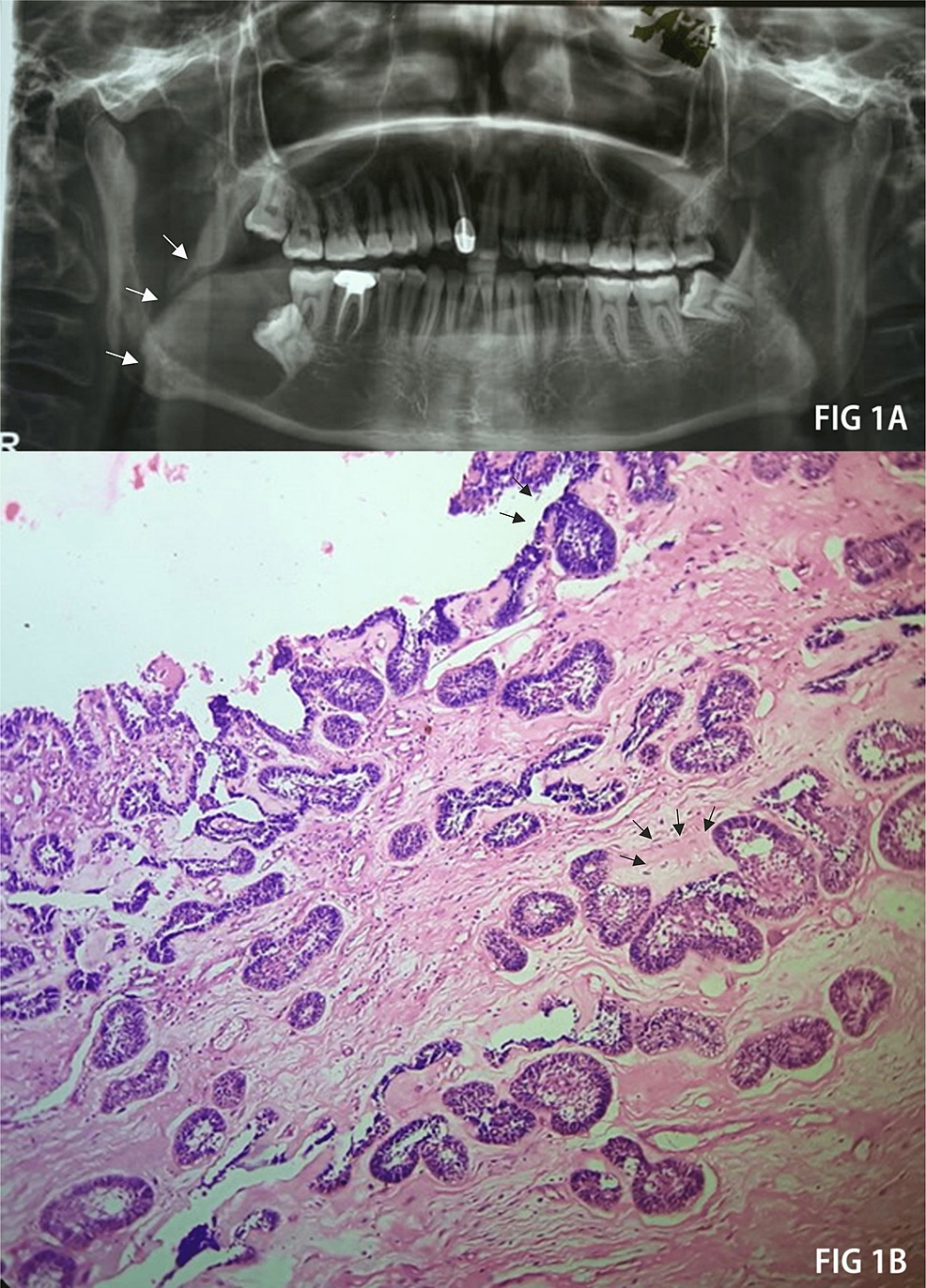
Preoperative radiographic investigations and histopathological presentation of the mural-type unicystic ameloblastoma. A (white arrows): A large radiolucent area on the right lower jaw. B (black arrow): Ameloblastoma islands present in the connective tissue stroma.

A large, radiolucent lesion that covered the majority of the right mandibular ramus and extended from the sigmoid notch neck of the condyle to the mandibular right second molar region was visible on the panoramic radiograph (Figure 1A). The lesion was associated with an impacted right third molar that was close to the distal root of the right second mandibular molar. The inferior alveolar nerve canal was displaced inferiorly. The medial cortical plates showed signs of perforation. The radiographic characteristics resembled odontogenic keratocyst, ameloblastoma, and dentigerous cyst. These can be their individual histopathological characteristics to distinguish them. On incision biopsy, under the microscope, a typical ameloblastomatous lining epithelium invaded the connective tissue wall, and there were islands of ameloblastoma in the connective tissue. Histopathology identified a unicystic ameloblastoma mural variant (Figure 1B).

**Figure 2:**
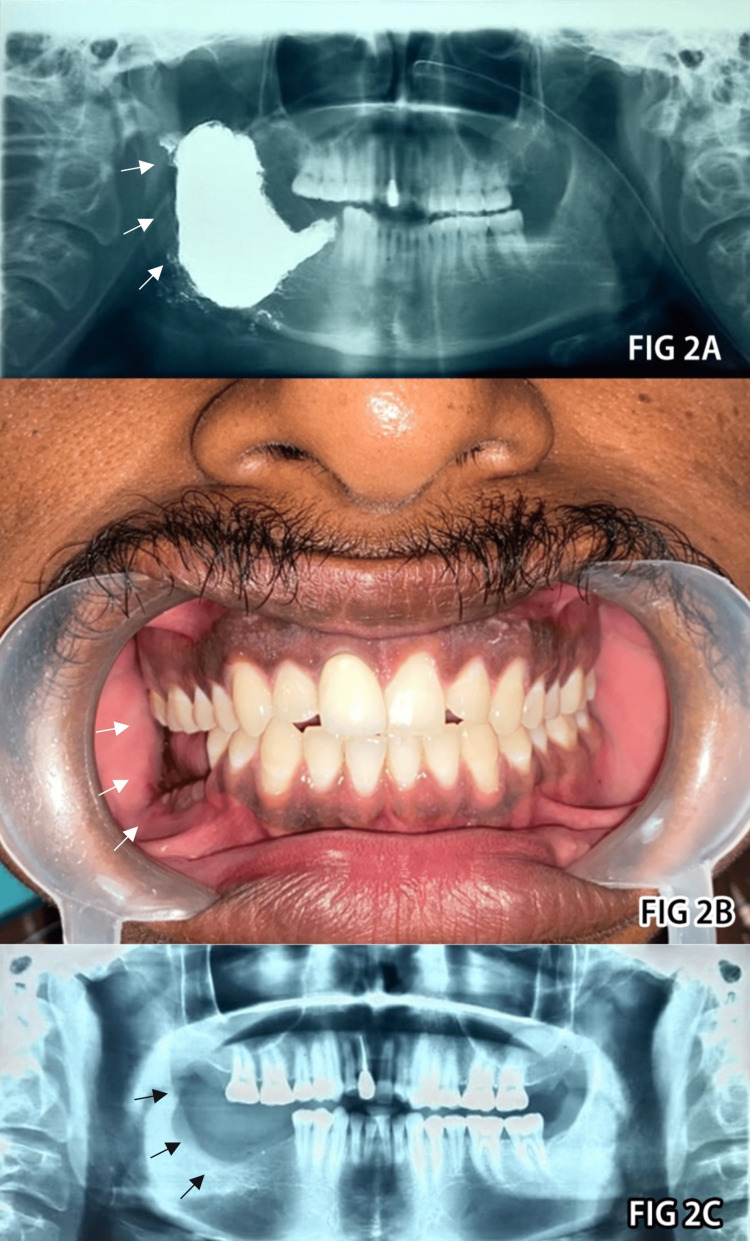
Postoperative radiographic and clinical presentation. A: A radiolucent area of filled iodoform gauze in immediate postoperative panoramic. B and C: The clinical and radiographic healing areas.

**Figure 3:**
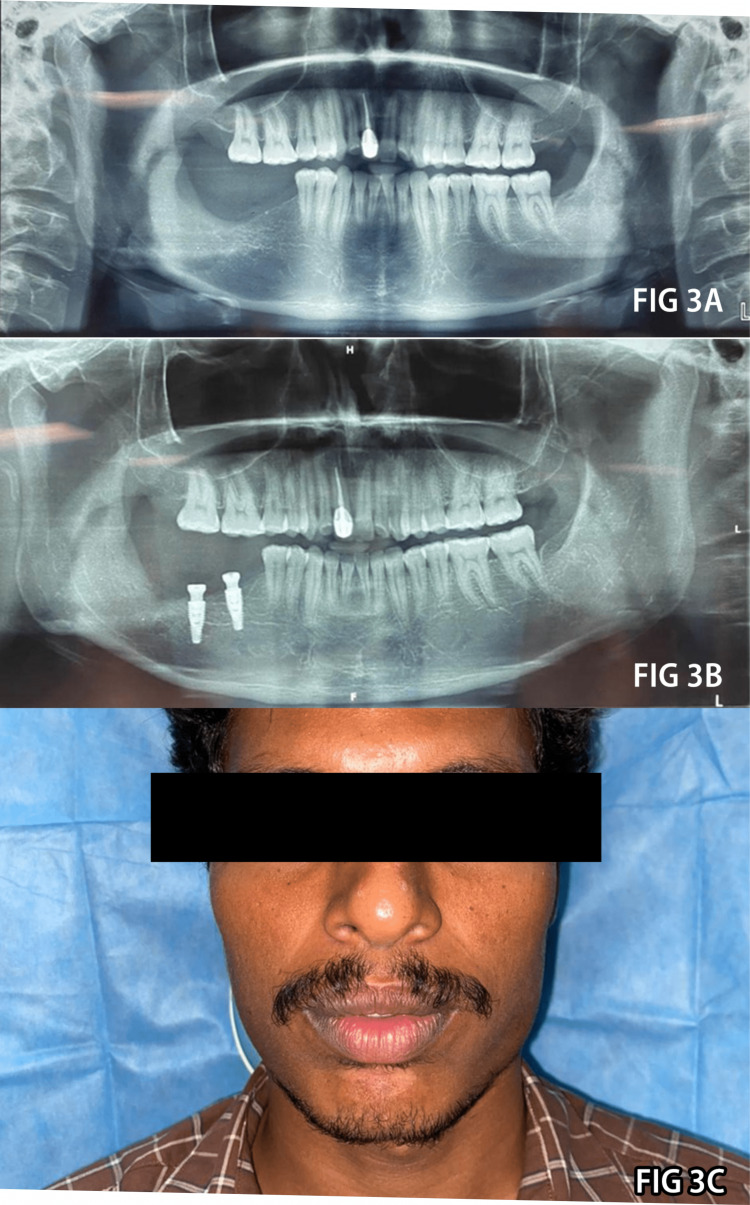
Rehabilitation phase. A: Panoramic radiographic image represents full bone regeneration after 24 months. B and C: Radiographic and clinical profile after implant placement.

The patient’s laboratory results were within acceptable ranges. Under general anesthesia, an oral approach was used, and an ascending ramus of the mandible incision was made in relation to the 47 and 48 teeth region. Beyond the boundaries of the cystic lesion, the elevated mucoperiosteal flap exposed the entire buccal and lingual cortex. The cyst was completely enucleated, and the lingual cortical plate, 48, 47, and 46 teeth were removed. Carnoy’s solution was then used to chemically cauterize the area. Petroleum jelly coating was done to protect the inferior alveolar nerve from Carnoy’s solution. To reduce dead space, the undercut bony margins of the cavity were removed and filled with iodoform gauze, and the surrounding tissues collapsed into the defect (Figure 2A). The wound was then closed. An extraoral compression bandage helped to adapt the soft tissues to the bony defect. Recovery from the surgery went smoothly. An adequate bone repair was seen after a year of regular clinical and radiographic monitoring (Figures 2B, 2C), and full bone regeneration was visible after 24 months (Figure 3, Panel A).

A fixed implant-supported prosthesis was envisioned for dental rehabilitation 30 months after the procedure. Two conventional dental implants were placed in regions 46 and 47 (Figure 3, Panel B). Temporary restorations were made following a second stage of surgery to ensure proper gingival collar growth after giving the wound enough time to heal without noticeable facial soft-tissue distortion (Figure 3, Panel C). Using the open tray technique and transfer coping with polyvinyl siloxane elastomeric impression material, the final impression was created. Regular monitoring of the patient revealed no signs of a recurrence as of this writing.

## Discussion

Three types of unicystic ameloblastomas are luminal, intraluminal, and mural. They are odontogenic epithelial tumors that comprise about 5% of all ameloblastomas. Mural unicystic ameloblastoma is an aggressive variant because it breaches the fibrous wall and has a high potential to invade the nearby cancellous bone [2]. Resection always results in significant tissue defects but has the lowest recurrence rate (3.6%) [3]. For the health-related quality of life, choosing the right reconstruction modality is equally important [4]. Farmand et al. [5] claim that the modified Brosch procedure is a cautious surgical alternative to resection or marsupialization. Lesioned teeth should be extracted, along with the infected soft tissues that overlie them and any partially eroded bone. This helps lower the likelihood of recurrence. It improves the procedure to be a better method for treating all cysts involving the ascending ramus of the mandible, allowing for quicker healing with bone regeneration and no visible facial soft-tissue distortion to visualize the cyst through full decortications. It can also be used to remove affected bony regions and overlying tissue. According to Christian et al. and Zemann et al., within a year of the patient receiving the procedure, ameloblastoma was successfully treated by segmental excision of the mandible with obturator implantation. They used dental implants for prosthetic rehabilitation during the postoperative period [6,7]. In this case report, the modified Brosch approach surgically removed the tumor with a rim of the involved bone while maintaining the continuity of the mandible. It provided an optimal supporting tissue bed for the prosthetic rehabilitation restoring function and esthetics, preserved the associated structures, and contributed to the patient’s perception of improved quality of life.

## Conclusions

The unicystic ameloblastoma mural variant has a high rate of recurrence. We treated the young adult in the current case using a modified Brosch procedure, which resulted in better bone regeneration. The modified Brosch procedure is an effective substitute for resection or marsupialization when treating large odontogenic lesions that extend to the molar, angle, and ramus regions of the mandible. With the least amount of repetition, this technique produced the best functional aesthetic results. There is little surgical morbidity associated with this strategy.
